# Digital Interventions and Mental Health Outcomes in Patients With Cancer: Systematic Review and Meta-Analysis

**DOI:** 10.2196/64754

**Published:** 2025-08-14

**Authors:** Zixuan Wu, Feifei Luo, Siyuan Wang, Xinyu Hu, Meifang Chen

**Affiliations:** 1 Bloomberg School of Public Health, Johns Hopkins University, Baltimore, MD, United States; 2 Division of Social Sciences, Duke Kunshan University, 8 Duke Avenue, Jiangsu, Suzhou, 215316, China, 86 051236657105; 3 Division of Natural and Applied Sciences, Duke Kunshan University, Suzhou, China

**Keywords:** digital intervention, mental health, cancer care, systematic review, meta-analysis, psychiatric, psychosocial burden, efficacy, subgroup analysis, qualitative, depression, anxiety, technologies, digital health, health informatics, digital literacy, PRISMA

## Abstract

**Background:**

Rising cancer rates have amplified psychiatric and psychosocial burdens, with 35%‐40% of patients exhibiting diagnosable psychiatric disorders. While digital mental health interventions (DMHIs) present potential solutions for improving emotional well-being in this population, evidence remains fragmented and lacks clarity regarding optimal implementation strategies. This study evaluates the efficacy of digital interventions on mental health outcomes in patients with cancer, with particular focus on intervention duration and stakeholder involvement as moderating factors.

**Objective:**

This study aims to (1) characterize digital interventions targeting mental health outcomes in patients with cancer; (2) quantify their effectiveness in reducing anxiety and depression; and (3) examine whether intervention duration and stakeholder involvement moderate treatment outcomes.

**Methods:**

This systematic review and meta-analysis followed the PRISMA (Preferred Reporting Items for Systematic Reviews and Meta-Analyses) statement guidelines and was retrospectively registered in PROSPERO on May 25, 2025 (CRD420251058005). A total of 8 databases (Cochrane Central Trials Registry, Web of Science, Scopus, PubMed, PsycINFO, Global Health, Embase, and Medline) were searched from inception to 2024. Eligible randomized controlled trials evaluated digital interventions for mental health in patients with cancer. Two reviewers independently screened studies, extracted data, and assessed risk of bias using the Cochrane Risk of Bias Tool 2.0. Random-effects meta-analyses calculated standardized mean differences (SMDs). Pooled results were reported as the odds ratio and 95% CI. The heterogeneity was assessed with the *I*² test (%). Subgroup analyses explored the potential effects of intervention duration and stakeholder involvement. Sensitivity analyses and publication bias assessments were performed to ensure the robustness of findings.

**Results:**

Twenty-two randomized controlled trials were included in the review. The geolocation involves 4 continents worldwide: Asia (n=9), Europe (n=5), North America (n=6), and Oceania (n=2). Interventions comprised meditation or mindfulness (n=3), education (n=8), self-management (n=11), physical exercise (n=4), and patient community communication (n=8). Twelve studies were included in the meta-analysis. Overall, digital interventions showed nonsignificant effects on depression (SMD −0.48, 95% CI −1.00 to 0.03; *P*=.07; 9 studies) or anxiety (SMD −0.61, 95% CI −1.29 to 0.06; *P*=.08; 8 studies) with substantial heterogeneity (*I*^2^>90%). Subgroup analyses revealed interventions (<1 month) significantly reduced anxiety (SMD −0.73, 95% CI −1.42 to −0.04; *P*=.04), while interventions (1‐2 months) reduced depression (SMD −0.18, 95% CI −0.35 to −0.01; *P*=.04). Interventions showed no statistically significant differences when stratified by stakeholder involvement. Sensitivity analyses excluding 1 outlier yielded significantly lower heterogeneity but preserved unchanged overall and subgroup patterns.

**Conclusions:**

While DMHIs overall showed no effect on anxiety or depression interventions, exploratory analyses suggest potential benefits of duration-tailored approaches. High heterogeneity and methodological limitations indicate that DMHIs may be most effective when integrated into personalized care models rather than standalone treatments. Future research should use standardized outcomes and investigate mechanisms underlying potential duration-dependent efficacy.

## Introduction

The global rise in cancer incidence has brought attention to the substantial burden of psychiatric and psychosocial consequences associated with the disease. Research on cancer populations in both Northern [1] and Southern Europe [2,3] affirms that 35%‐40% of patients with cancer exhibit diagnosable psychiatric disorders, as determined by the International Statistical Classification of Diseases and Related Health Problems 10th Revision psychiatric interview [4]. These include stress-related, neurocognitive, adjustment, and somatic symptom disorders, as well as neuropsychiatric manifestations, including psychosis caused by psychiatric changes associated with paraneoplastic syndromes and drug-related psychiatric symptoms [5], which significantly jeopardize patient engagement in cancer treatment, thus affecting both mental and physical health outcomes. Among these conditions, the prevalence of anxiety among patients with cancer is up to 10%, regardless of the treatment phase or point in the disease trajectory [5]. Depression emerges as another prevalent comorbid mental disorder accompanying cancer, with patients reporting symptoms of mild (27.2%) or moderate depression (22%), as well as major depression (18%) [6]. This demonstrates a significant demand for psychological and mental health services among patients with cancer, leading to urgent calls for the development of multisectoral mental health care approaches, especially those targeting anxiety and depression.

For cancer survivors, studies examining anxiety and depression found that 40% experienced moderate to high anxiety, and approximately 20% reported moderate to high depression [7]. Echoing this, another study reported that 19% of posttreatment cancer survivors have clinically significant depression [8], with a systematic review concluding that the estimated pooled prevalence of anxiety among cancer survivors is 21% [9]. Beyond individual symptoms, factors such as survivors’ occupation, surgery type, comorbidities, and age were all found to contribute to this increasing psychological burden, indicating cancer survivors’ need for long-term psychological support [10]. However, the detection and treatment of depression and anxiety in patients with cancers frequently fall short, due to overlapping symptoms with the fatigue and pain associated with cancer itself [11]. This pattern emphasizes the prolonged psychological impact and the importance of monitoring and addressing mental health in survivorship care plans.

Digital mental health interventions (DMHIs) emerge as a highly promising care model, offering substantial potential as a scalable solution to bridge the service provision gap. These technology-based approaches encompass various forms, including mobile apps, web-based programs, virtual reality, wearable devices, and video games, enabling the fusion of information technology with theory-based mental health interventions [12]. For example, internet and computer-based delivery formats can enhance access to cognitive behavior therapy (CBT). Computerized CBT delivered over the internet or via computer in clinical settings implements CBT principles through structured lessons, often accompanied by homework assignments and supplementary materials [13]. Additionally, mHealth apps can function as standalone self-help programs or as adjunctive treatment modalities within guided programs incorporating components like cognitive therapy, behavioral activation, psychoeducation, or symptom monitoring [14]. Online communities also serve as platforms for receiving anonymous emotional support [15]. The anonymity, immediate access, and flexibility afforded by DMHIs effectively overcome common barriers to accessing care, such as cost, time constraints, or stigma [16]. They empower users to gain knowledge about their health, connect with others facing similar challenges, shift personal attitudes regarding health, track specific conditions, identify health goals, make informed treatment choices, and enhance communication with health care providers.

While research has demonstrated the effectiveness of DMHIs for general mental health treatment, their application in specific clinical domains such as oncology remains underexplored. Given the encouraging potential of DMHIs and the high comorbidity of depression and anxiety in patients with cancer throughout various treatment stages and disease progression, there is a clear opportunity to expand their application. Recent evidence suggests promising applications in oncology that digital health tools can empower patients with cancer by fostering autonomy and self-acceptance, aiding in environmental navigation, nurturing social connections, and pursuing life aspirations [14,17,18]. Among seven trials using mobile apps for patients with cancer, 3 reported reduced anxiety, and 4 demonstrated decreased fatigue, lower symptom distress, and reduced sleep disturbance [19-25]. Additional studies of various digital modalities—including text messages, video conferences, psychoeducational programs, and online group discussions—have shown increased quality of life and reduced anxiety compared to traditional interventions alone [20,26-28].

Despite promising findings from individual studies, only a limited number of reviews have examined the effectiveness of DMHIs in improving mental health outcomes for patients with cancer [14,17]. One meta-analysis collected data before 2020 to examine this evidence, but its findings may be outdated due to rapid advances in DMHIs in recent years [29]. Additionally, the analysis highlighted that insufficient data on how often people actually use these interventions makes it difficult to understand their true effectiveness. The researchers recommended that future studies should systematically examine what factors influence both whether people engage with these interventions and how well they work for this population [29]. Moreover, it emphasizes that limited data on intervention uptake obscures the understanding of effectiveness, while research should systematically address factors influencing the intervention uptake and its outcomes across this group [29].

To address this knowledge gap and justify the need for developing psychosocial care strategies for patients with cancer, this systematic review and meta-analysis aims to (1) summarize current DMHIs among patients with cancer updated till 2024, (2) evaluate their pooled effects on mental health outcomes and associations between DMHIs and mental health improvements, thereby consolidating fragmented findings from individual studies into a methodologically robust synthesis, and (3) conduct an exploratory subgroup analysis by intervention duration, type, and targeted outcomes. We will specifically analyze intervention duration and stakeholder participation across different time intervals, targeting both individual and group levels, to identify optimal strategies for improving mental health outcomes. These subgroup analyses address fundamental clinical questions about optimal intervention design: duration-based analysis is grounded in psychological theory that anxiety and depression require different therapeutic timeframes [5], while stakeholder involvement classification examines whether peer support enhances effectiveness in cancer populations where social isolation is prevalent [3,8]. Understanding these intervention characteristics is essential for resource allocation and program design in oncology settings, making these exploratory analyses valuable despite limited study numbers. These findings will inform the development of improved mental health management models that promote psychological well-being throughout cancer survivorship.

## Methods

### Protocol Registration

This systematic review and meta-analysis followed the PRISMA (Preferred Reporting Items for Systematic Reviews and Meta-Analyses) statement guidelines (Multimedia Appendix 1) [30]. The protocol was retrospectively registered in PROSPERO on May 25, 2025 (420251058005).

### Study Design

In the preliminary stage of this review, mental health outcomes were broadly defined to include not only clinical measures of psychological symptoms (eg, depression, anxiety, distress, and stress), but also psychological well-being (eg, quality of life and emotional functioning) and psychological resources (eg, self-efficacy and resilience). This inclusive approach reflects contemporary understanding of mental health in oncology settings, that patients with cancer experience a spectrum of psychological responses that extend beyond clinical diagnoses, where both symptom reduction and enhancement of coping resources are considered important therapeutic targets [31]. However, during the meta-analysis stage, we focused exclusively on anxiety and depression outcomes due to the limited number of studies examining other mental health conditions.

Randomized controlled trials (RCTs) that allocated patients with cancer to receive treatment with digital interventions versus no treatment or traditional interventions were eligible for inclusion. We considered trials to be eligible if they included patients with documented cancer diagnosis and were original or secondary analyses of RCT evaluations of digital interventions whose mental health outcomes were included. Patients with cancer were defined as individuals diagnosed with any type of cancer, irrespective of disease stage, treatment phase, type of treatment, and time since diagnosis. Only digital interventions that delivered manualized therapeutic content to users via a digital platform (eg, smartphone, tablet, and computer) were included.

Studies were excluded if they were other types of studies (eg, observational, review, protocol, and case reported), not peer-reviewed journal articles (eg, dissertations and conference presentations), written in a language other than English, without full text or if they did not reveal the effectiveness of digital intervention on patient mental health outcomes. No restrictions were placed on the target population, type of cancer, type of mental health condition targeted, or the language in which the interventions were delivered.

### Searching and Selection Method

To find eligible RCTs, a comprehensive search strategy was developed in consultation with a health sciences librarian at Duke University, United States specializing in systematic reviews. After several rounds of discussions for the searching strategy between our research group, the independent investigator (ZW) searched the Cochrane Central Trials Registry, Web of Science, Scopus, PubMed, PsycINFO, Global Health, Embase, and Medline by using a comprehensive searching strategy (Multimedia Appendix 2). ZW, XH, SW, and FL then exported the retrieved studies to Covidence to identify and remove duplicates. After that, ZW, XH, SW, and FL screened the titles and abstracts together. ZW and SW independently screened the full texts of studies included from the second step. XH resolved any disagreements between the 2 reviewers in the second and third steps.

### Data Extraction and Assessment of Methodological Quality

The following information was extracted from each publication using Microsoft Excel (Microsoft Corporation) spreadsheet [32]: study characteristics (country of origin, year of publication, aim, and sample size), population characteristics (age, gender, and type of cancer), intervention characteristics (content, duration, and frequency), and outcome measures (mental health outcomes). Special attention was given to identifying the primary mental health outcomes targeted by each intervention, as these would form the basis for subsequent meta-analysis. The first author independently extracted the data, and another author checked the data extraction to determine interrater reliability. Consensus was reached through discussion. The data extraction form was used to complete a narrative synthesis of the results. Additionally, evidence regarding the effects of digital interventions on mental health among patients with cancer was synthesized by collating the publication details, study designs, participants, intervention types, duration, samples, and results.

### Risk of Bias Assessment

In addition, the risk of bias was assessed using Version 2 of the Cochrane risk-of-bias tool for randomized trials (RoB 2) [33]. The RoB 2 comprises 5 domains and an overall risk domain in RCTs: randomization process, deviations from intended interventions, missing outcome data, measurement of the outcome, and selection of the reported result, each of which was scored against a 3-point rating scale corresponding to a “low,” “some,” or “high” risk of bias. Ratings were independently conducted by 3 coders (ZW, SW, and XH). Discrepancies were resolved through mutual discussion.

### Data Analysis

We used both narrative and statistical methodologies to consolidate the extracted data. Initially, in narrative synthesis, we used texts and tables to delineate the characteristics of the included studies, encompassing details about the population, intervention, and outcome measures. Subsequently, we categorized and encapsulated the findings of the included studies based on the type of mental health outcomes, duration of interventions, and stakeholders involved.

For the meta-analysis, we focused specifically on 2 primary mental health outcomes, that is, depression and anxiety, as these were the most consistently reported across studies and represent significant psychological challenges for patients with cancer. Studies were grouped for meta-analysis if they reported quantitative data on either of these 2 outcomes using validated measurement instruments. Additionally, we conducted separate meta-analyses for each of these mental health outcomes, with additional subgroup analyses examining the influence of intervention duration and stakeholder involvement on treatment effects. The process was conducted if at least 2 studies examining the same mental health outcome provided sufficient data (mean, SD postinterventions, and number of participants in each intervention group). Data synthesis was performed using Revman Manager 5.3 (the Review Manager software 5.3, The Nordic Cochrane Center, The Cochrane Collaboration), and all numeric outcome data were double entered to prevent data entry errors. We measured the effect of each trial and the overall effect using the standardized mean difference (SMD; Cohen *d*) because the outcome data (severity of depressive symptoms) were continuous, and the scales we included in the studies used to measure the outcome were different. Heterogeneity was quantified using *I*² statistics, and a chi-square *P* value≤.05 indicates heterogeneous meta-analyzed studies. When *I*² <50%, a fixed effects model was used. A random effects model was applied where there was evidence of significant heterogeneity (*I*² ≥50%). For data extracted measured by different scales and that were continuous, the SMD was used [32]. Heterogeneity thresholds were defined for *I*² of 25% (low), 50% (heterogeneity), and 75% (high heterogeneity) [34]. Publication bias was assessed using funnel plots and Egger test for the overall meta-analyses, though formal publication bias assessment was not feasible for subgroup analyses due to insufficient study numbers (fewer than 10 studies per subgroup) [34-36].

### Sensitivity Analysis

To assess the robustness of our findings and examine the influence of individual studies on the overall results, we conducted sensitivity analyses using a leave-one-out approach in R (version 4.3.1; R Foundation for Statistical Computing) for both depression and anxiety outcomes. This involved systematically removing 1 study at a time from the meta-analysis and recalculating the pooled effect size to determine whether the findings were driven by any single study and helped identify potential sources of heterogeneity in the observed results.

## Results

### Study Characteristics

**Figure 1.**
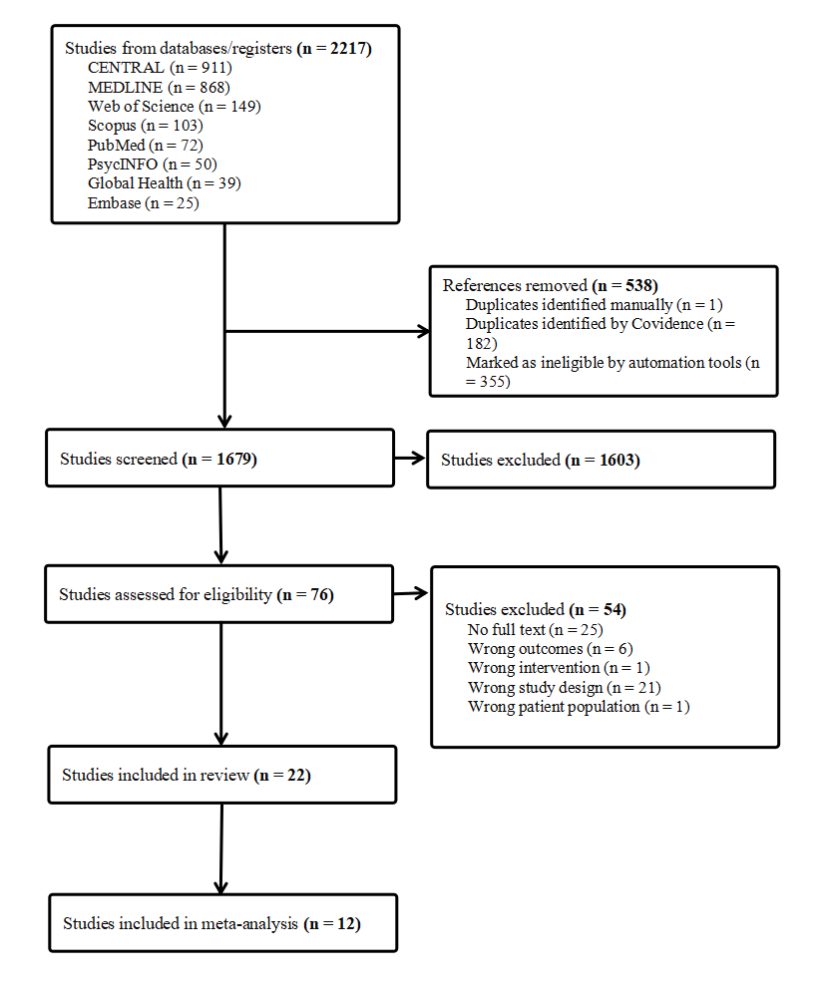
PRISMA (Preferred Reporting Items for Systematic reviews and Meta-Analyses) diagram for study selection process.

After systematically searching 8 electronic databases, a total of 2217 studies were identified and selected. Following deduplication and screening, 22 studies met our inclusion criteria, with 12 of these included in the meta-analysis. Two studies were excluded from quantitative synthesis due to combined reporting of depression and anxiety outcomes and insufficient postintervention data [21,28]. The PRISMA diagram details the flow of selection in Figure 1.

The detailed information of the included studies, with a total of 2912 patients, is shown in the Multimedia Appendix 3 [18,19,21,22,25,26,28,37-51]. The sample sizes ranged from 60 to 352 patients. The geolocation involves 4 continents worldwide: Asia (n=9), Europe (n=5), North America (n=6), and Oceania (n=2). Most studies (n=13, 59.1%) focused on patients with breast cancer [18,22,25,26,37-45], while the remainder addressed myeloproliferative neoplasms [46]; 1 was related to upper gastrointestinal cancers [47]. The other 7 studies were related to patients with cancer in general [19,21,28,48-51]. The studies were all published from 2014‐2024, with a rising trend overall and a peak publication year of 2019 (n=6), indicating growing interest in DMHIs for populations with cancer.

### Characteristics of Interventions

Digital interventions varied considerably in their approach, content, and delivery format. The intervention contents fell into 5 main categories: meditation or mindfulness (n=3), education (n=8), self-management support (n=11), physical exercise (n=4), and communication within patient communities (n=8). The intervention duration ranged from 4 weeks to 6 months (mean duration: 10.7 weeks, and mostly was less than 3 months). Based on stakeholder involvement, 16 studies are individual-based centered on mindfulness, individual self-management reminders, and education, while 6 studies include social group-based interventions that fulfill one’s social needs and peer support functions. For instance, 1 study used a group-based intervention in which participants joined an app-based community to track daily steps and compare their rankings with peers [39]. Compared to the control group in which the app was only used for steps tracking, the aim of the intervention was to create a sense of belonging among participants, which is of the same logic as other studies using social group-based intervention [37,40,46,48,51]. In studies using individual self-management reminders to intervene, the reminder usually focuses on 1 specific aspect of a patient’s life, such as the diet, the medicine adherence, or symptoms [19,22,23,38,42-44,47,50,52]. One example is a comprehensive lifestyle management intervention, where participants used an app to (1) schedule appointments and track important dates, (2) monitor health indicators (eg, pain, fatigue, and sleep patterns), (3) maintain social connections with friends and family, and (4) access weekly educational resources and local event information [50]. In cases where physical training was conducted, patients were asked to watch face-to-face video instructions multiple times a week to do muscle or cardiorespiratory capacity training, at the same time, receiving cancer rehabilitation knowledge [38].

### Control Interventions

Control groups primarily received usual care (n=16), standard care (n=1), or other control therapy (eg, a traditional rehabilitation for physical exercise; n=1), and health education or information track only (n=4). In this review, usual care and standard care included routine nursing.

### Variables, Instruments, and Results

The included studies targeted various mental health outcomes, with anxiety (n=8, 36.4%), depression (n=9, 40.9%) being the most frequently assessed. Other outcomes included emotional well-being or functioning (n=5), stress (n=2), self-efficacy (n=2), posttraumatic growth (n=1), psychological resilience (n=1), and mood (n=1).

Across studies, 19 different validated instruments were used to measure these outcomes. In the selected studies, mental health was assessed using various scales as follows: National Institutes of Health Patient-Reported Outcomes Measurement Information System [46], Functional Assessment of Cancer Therapy-General and Short-Form 12-item Health Survey questionnaire [50], SF-36 [38], Health Related Quality of Life and 36-item Short Form Self Survey [49]. The emotional function was assessed using the Short Form-36 item Health Survey questionnaire [43], Functional Assessment of Cancer Therapy-Breast [42], and the European Organization for Research and Treatment of Cancer Quality of Life Questionnaire [22,37,47]. In some cases, depression and anxiety were assessed together using the Hospital Anxiety and Depression Scale [18,19,21,28,45,51] and the Depression Anxiety and Stress Scale-21 [25]. In other cases, depression was assessed using the Center for Epidemiologic Studies Depression Scale [41], Beck Depression Inventory [40], and Patient Health Questionnaire-8 [48], while anxiety was assessed using National Comprehensive Cancer Network Distress Thermometer [51], the Spielberger State-Trait Anxiety Scale (State-Trait Anxiety Inventory) [40], the Hamilton Anxiety Rating Scale [20], and the State-Trait Anxiety Inventory [26]. Mood was assessed using the Hospital Anxiety and Depression Scale [18]. Stress was assessed using the Depression Anxiety Stress Scale-21 [25] and the Perceived Stress Scale [49]. Distress was assessed using the National Comprehensive Cancer Network Distress Thermometer [28,39,51]. Self-esteem was assessed using the Standard Inventory of Cancer Patient Adjustment [40] and the Rosenberg Self-Esteem Scale [26,45]. Posttraumatic growth was assessed using the 21-item Posttraumatic Growth Inventory [46] while psychological resilience was assessed by the Connor-Davidson Resilience Scale [51].

### Methodological Quality of Trials

**Figure 2.**
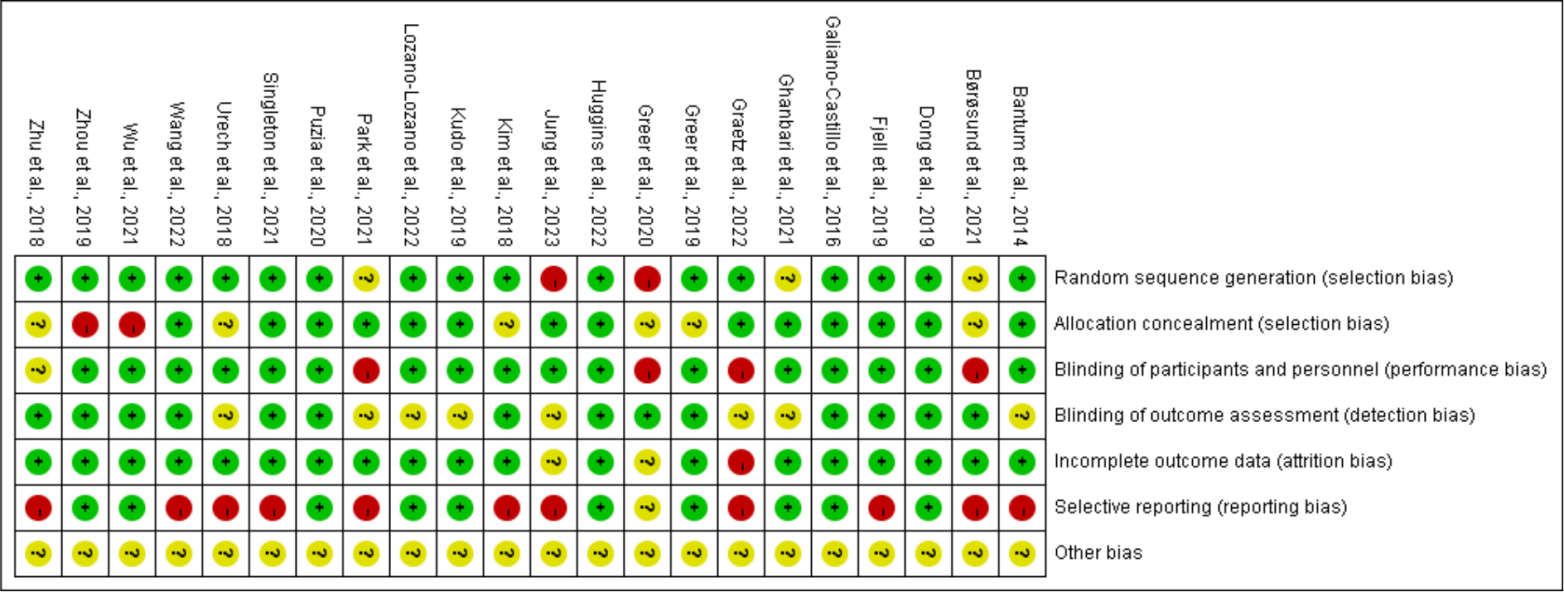
Risk of bias among included trials.

Assessment of the methodological quality of studies on the Cochrane Risk of Bias 2.0 tool found most studies to have some level of potential bias (Figure 2). Three studies have been classified as low risk, 4 studies as moderate risk, and the remaining studies are classified as high risk. Selective reporting was identified to be the largest source of bias, with 11 studies (50%) not reporting accurate information due to their self-reported characteristics in these studies. Blinding of participants and personnel was the second most common source of potential bias, with 4 studies (18.2%) reporting that study participants were partially aware of the intervention received by study participants. In addition to the bias mentioned, most studies had sound random sequence generation, allocation concealment processes, blinding of outcome assessment, and used analytical techniques to minimize bias in missing postintervention outcome data.

### Meta-Analysis Outcomes

**Figure 3.**
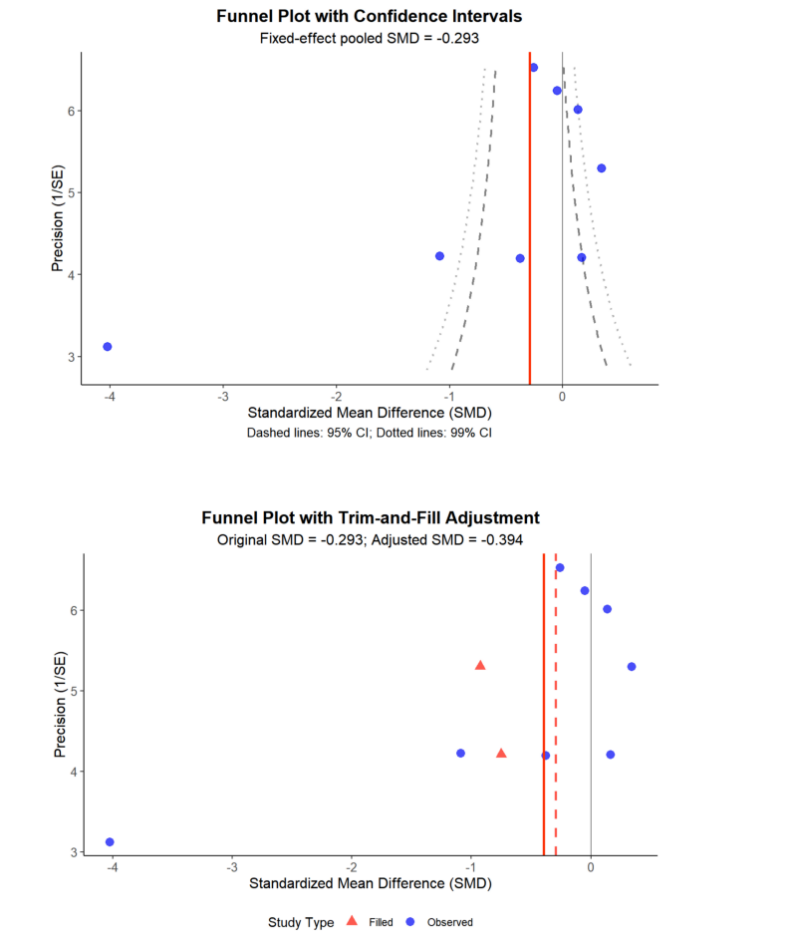
Funnel plots for anxiety before and after trim-and-fill adjustments. SMD: standardized mean difference.

Substantial heterogeneity was observed in the primary analyses, prompting additional assessment of publication bias through funnel plots, Egger regression tests, and trim-and-fill procedures. Due to the limited number of studies within subgroups, these assessments were not extended to subgroup analyses as they would lack statistical power. The complete inventory of publications included in the meta-analysis is documented in Multimedia Appendix 4. Forest plots depicting overall effect sizes and subgroup analyses, and the funnel plots for overall effects are presented in Figures3  4.

**Figure 4.**
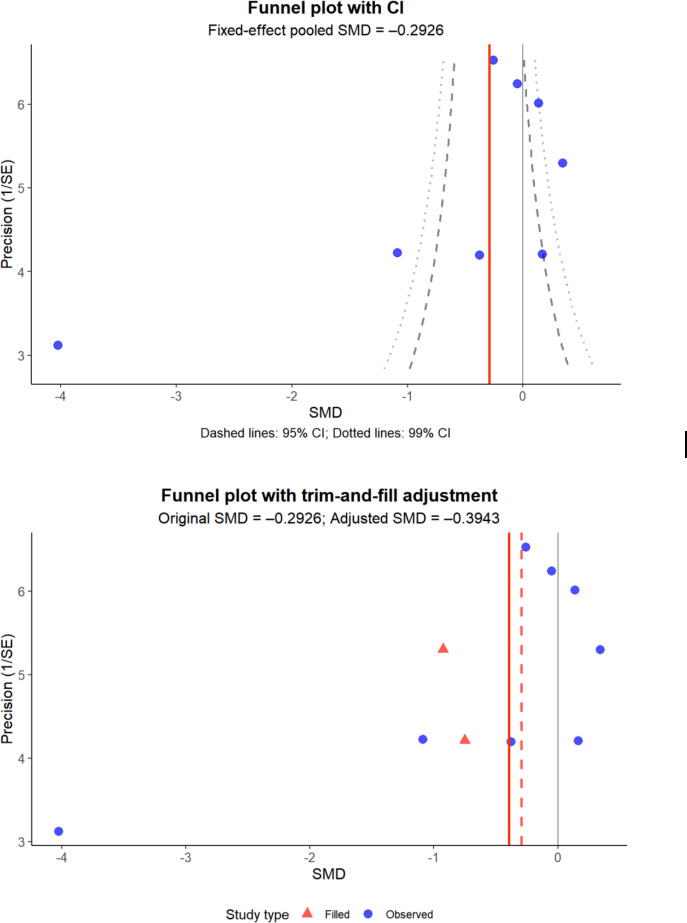
Funnel plots for depression before and after trim-and-fill adjustments.

### Main Analyses on Anxiety

**Figure 5.**
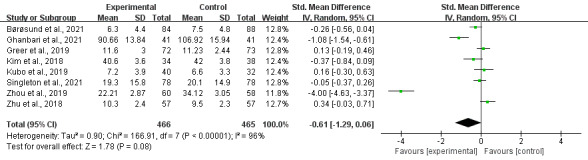
Digital intervention overall effects on anxiety [19,25,26,40,44,45,49,51].

Results of 8 studies including 931 participants were meta-analyzed as shown in Figure 5. No statistically significant difference (*P*=.08) in the severity of anxiety symptoms was found between the intervention group and usual care group (SMD −0.61, 95% CI −1.29 to 0.06), with substantial heterogeneity in the evidence (*P*<.0001; *I*^2^=96%).

Assessment of publication bias using the Egger test for funnel plot asymmetry revealed significant asymmetry suggesting potential publication bias (Figure 3). The funnel plot shows an asymmetric distribution of studies around the pooled effect estimate (SMD−0.293), with several studies appearing outside the 95% confidence intervals. The Duval and Tweedie trim-and-fill analysis identified potentially missing studies and adjusted the pooled effect size from the original SMD =−0.293 to an adjusted SMD =−0.394. This adjustment suggests that publication bias may have underestimated the true treatment effect, with the corrected analysis indicating a slightly larger beneficial effect of DMHIs.

### Main Analysis on Depression

**Figure 6.**
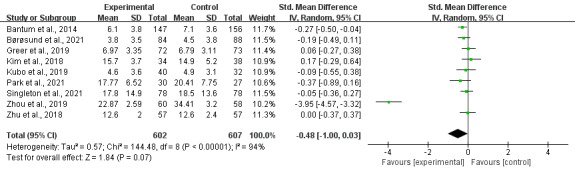
Digital intervention overall effects on anxiety [19,25,40,41,44,45,48,49,51].

Nine studies assessed the effect of digital interventions on depression, including 1209 participants. The pooled data showed no significant difference in depressive reduction between the intervention and control groups (SMD −0.48, 95% CI −1.00 to 0.03; *P*=.07) with a high heterogeneity of 94% (Figure 6).

Similar to findings on anxiety, assessment of publication bias using the Egger test for funnel plot asymmetry revealed significant asymmetry (Figure 4), suggesting potential publication bias. The significant intercept suggests that smaller studies with nonsignificant or less favorable results may be missing from the literature. The Duval and Tweedie trim-and-fill analysis estimated few potentially missing studies and adjusted the pooled effect size from 0.055 to 0.221 (95% CI 0.115-0.328). This substantial change in effect size suggests that publication bias may have inflated the observed treatment effect.

### Subgroup Analyses

Given the substantial heterogeneity observed in the overall meta-analysis, subgroup analyses were conducted to explore how intervention duration and stakeholder involvement influence the effectiveness of digital interventions. The analyses were organized by primary outcome (anxiety and depression) with subgroup comparisons within each condition.

### Digital Intervention Effects on Anxiety: By Intervention Duration

**Figure 7.**
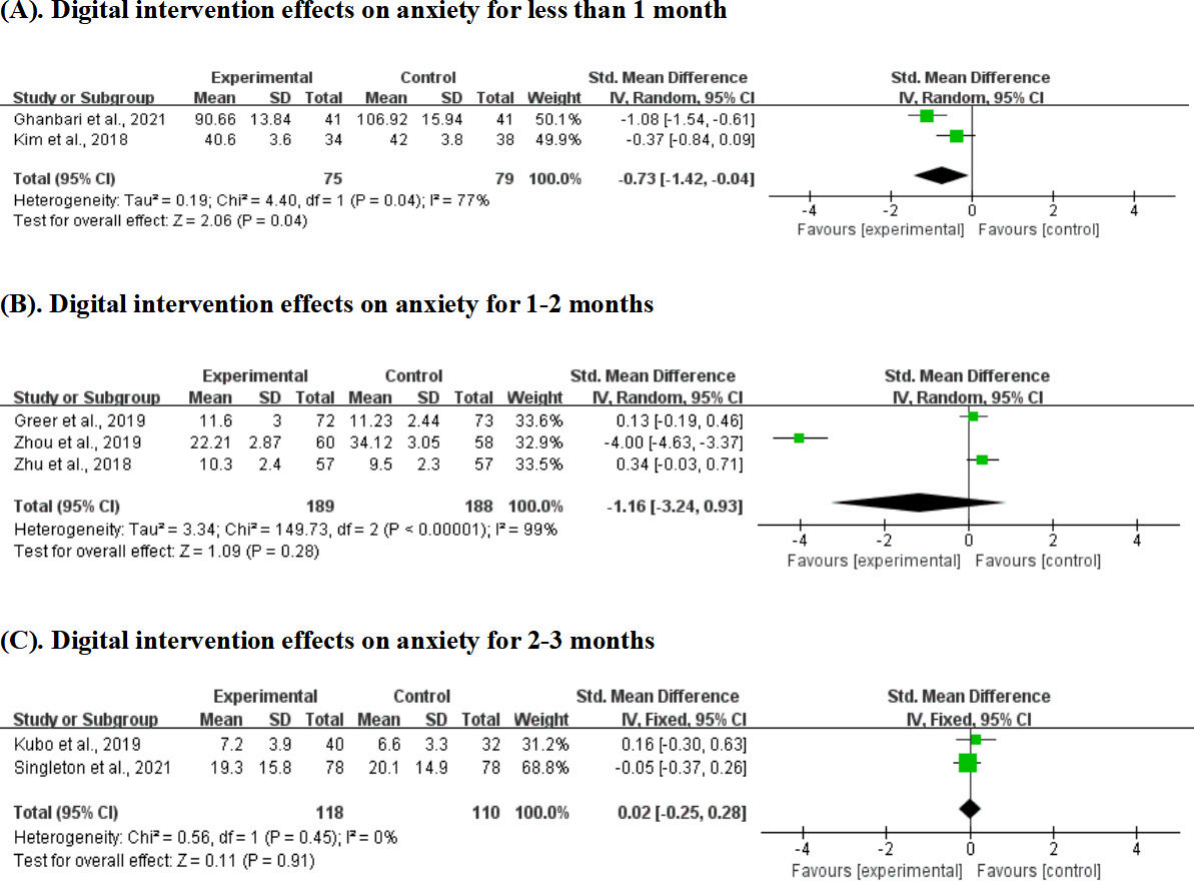
Effect sizes of the digital intervention group versus the control group on anxiety for various durations of time. (A) Digital intervention effects on anxiety for less than 1 month, (B) digital intervention effects on anxiety for 1-2 months, and (C) digital intervention effects on anxiety for 2-3 months [19,25,26,40,44,45,51].

Short-term interventions (<1 month) demonstrated the strongest evidence for anxiety reduction, with significant benefits observed across 2 studies involving 154 participants (SMD −0.73, 95% CI −1.42 to −0.04; *P*=.04). However, the evidence quality was moderate due to considerable heterogeneity (*I*²=77%). Medium-term interventions (1‐2 months) showed no significant effect on anxiety across three studies with 377 participants (SMD −1.16, 95% CI −3.24 to 0.93; *P*=.28), with extremely high heterogeneity (*I*²=99%) indicating substantial variation in intervention characteristics or implementation. Longer-term interventions (2‐3 months) similarly failed to demonstrate significant anxiety benefits across 2 studies with 228 participants (SMD 0.02, 95% CI −0.25 to 0.28; *P*=.91) with low heterogeneity (*I*²=0%; Figure 7).

### Digital Intervention Effects on Anxiety: By Stakeholder Involvement

**Figure 8.**
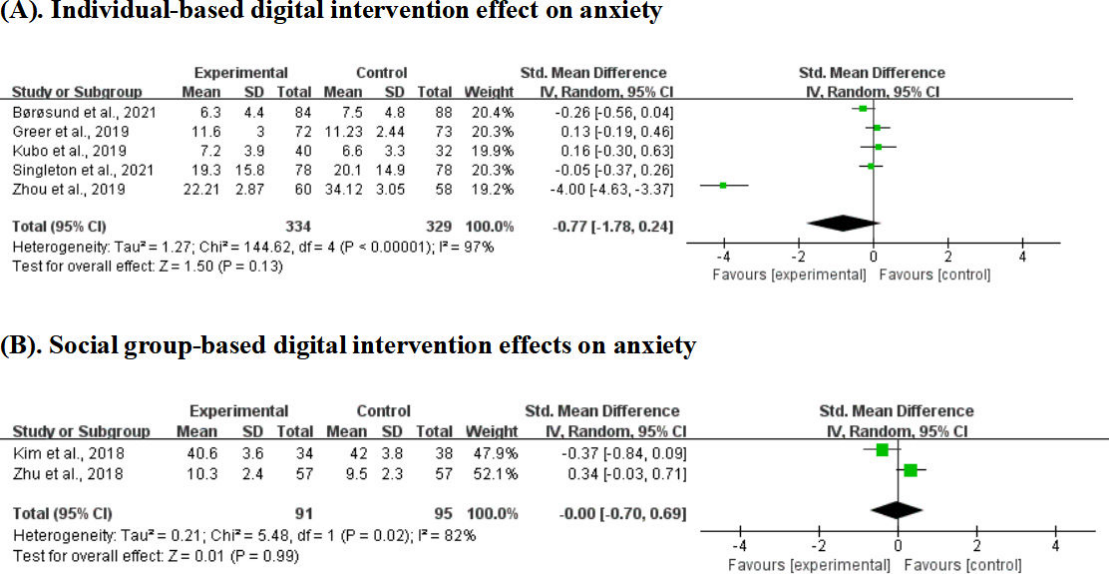
Effect sizes of the individual-based digital intervention group versus the control group on anxiety. (A) Individual-based digital intervention effects on anxiety and (B) social group-based digital intervention effects on anxiety [19,25,40,44,45,49,51].

Individual-based interventions for anxiety showed no significant overall effect across 5 studies with 663 participants (SMD −0.77, 95% CI −1.78 to 0.24; *P*=.13), despite the larger sample size. High heterogeneity persisted (*I*²=97%). Social group-based interventions for anxiety demonstrated no effect across 2 studies with 186 participants (SMD −0.00, 95% CI −0.70 to 0.69; *P*=.99), with high heterogeneity (*I*²=82%; Figure 8).

### Digital Intervention Effects on Depression by Intervention Duration

Short-term interventions (<1 month) showed no significant effect on depression across 2 studies with 129 participants (SMD −0.08, 95% CI −0.61 to 0.45; *P*=.76), contrasting with their effectiveness for anxiety outcomes.

**Figure 9.**
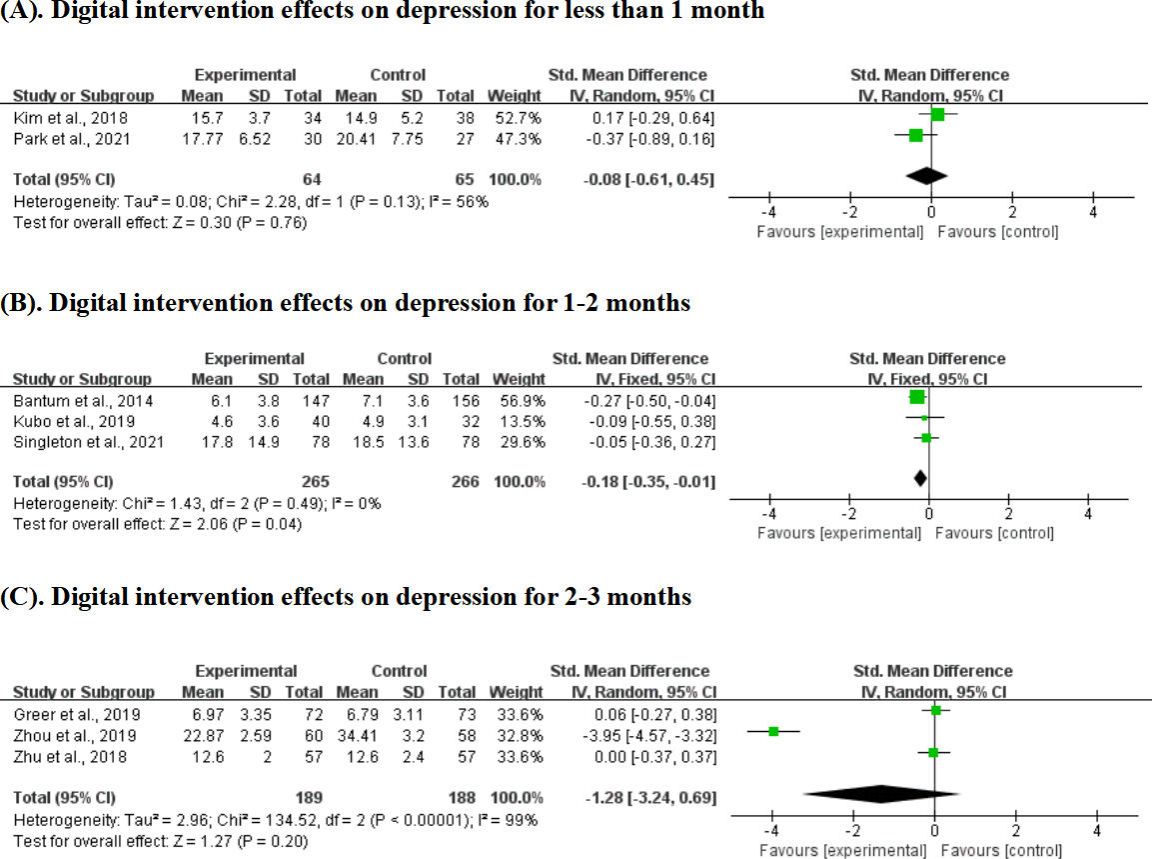
Effect sizes of the digital intervention versus the control group on depression for various durations. (A) Effects on depression for <1 month, (B) effects on depression for 1-2 months, and (C) effects on depression for 2-3 months [19,25,40,41,44,45,48,51].

Medium-term interventions (1‐2 months) demonstrated significant depression reduction across 3 studies with 531 participants with minimal heterogeneity (SMD −0.18, 95% CI −0.35 to −0.01; *P*=.04; *I*²=0%). Longer-term interventions (2‐3 months) failed to show significant benefits for depression across 3 studies with 377 participants (SMD −1.28, 95% CI −3.24 to 0.69; *P*=.20), with high heterogeneity (*I*²=99%; Figure 9).

### Digital Intervention Effects on Depression by Stakeholder Involvement

**Figure 10.**
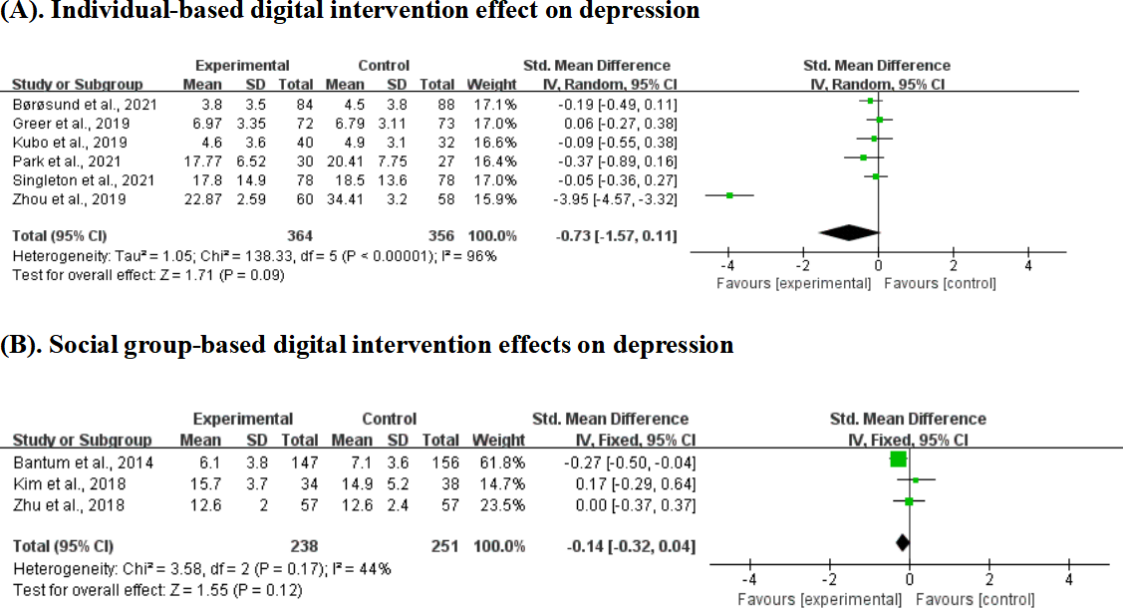
Effect sizes of the individual-based digital intervention group versus the control group on depression. (A) Individual-based digital intervention effects on depression. (B) Social group–based digital intervention effects on depression [19,25,40,41,44,45,48,49,51].

Individual-based interventions for depression showed no statistically significant effect across 6 studies with 720 participants (SMD −0.73, 95% CI −1.57 to 0.11; *P*=.09). This analysis included the largest sample size but exhibited high heterogeneity (*I*²=96%). Social group-based interventions for depression demonstrated no significant effect across 3 studies with 489 participants (SMD −0.14, 95% CI −0.32 to 0.04; *P*=.12), with moderate heterogeneity (*I*²=44%; Figure 10).

### Sensitivity Analyses

Sensitivity analyses using a leave-one-out approach revealed that findings for both anxiety and depression were influenced by individual studies, with maximum changes in effect sizes of 0.48 and 0.45, respectively, both attributed to Zhou et al (2019) [44]. While most studies demonstrated modest influence on the overall results, the presence of high-influence studies in both analyses suggests that the findings should be interpreted with caution. Nevertheless, the overall effect direction remained unchanged, and heterogeneity remained high for both outcomes. Subgroup analyses demonstrated similar patterns, with statistical significance unchanged and no substantial differences in heterogeneity categorization. Moreover, the result would not be documented due to the limited number of included studies for subgroups. The detailed results of the sensitivity analysis for overall effects are presented in Table 1.

**Table 1.** Sensitivity results of anxiety and depression groups for the overall effects.

Studies excluded	SMD^a^	Lower CI	Upper CI	*P* value	*I*² (%)	Change in SMD
Anxiety						
Zhou et al [44], 2017	−0.15	−0.49	0.19	.38	82.50	0.48
Zhu et al [45], 2018	−0.77	−1.86	0.31	.16	98.11	−0.14
Kubo et al [51], 2019	−0.75	−1.84	0.35	.18	98.27	−0.11
Greer et al [19], 2019	−0.74	−1.84	0.36	.19	98.09	−0.11
Singleton et al [25], 2021	−0.72	−1.83	0.40	.21	98.11	−0.09
Ghanbari et al [26], 2021	−0.57	−1.69	0.55	.32	98.33	0.06
Børøsund et al [49], 2021	−0.69	−1.81	0.44	.23	98.11	−0.06
Kim et al [40], 2018	−0.67	−1.79	0.46	.24	98.36	−0.04
Depression						
Zhou et al [44], 2017	0.09	−0.22	0.39	.58	82.90	0.45
Bantum et al [48], 2014	−0.54	−1.48	0.40	.26	97.80	−0.172
Greer et al [19], 2019	−0.43	−1.44	0.59	.41	98.30	−0.06
Kim et al [40], 2018	−0.44	−1.44	0.57	.39	98.40	−0.07
Kubo et al [51], 2019	−0.41	−1.42	0.61	.43	98.50	−0.04
Park et al [52], 2021	−0.37	−1.39	0.64	.47	98.50	−0.00
Singleton et al [25], 2021	−0.41	−1.43	0.60	.43	98.30	−0.04
Børøsund et al [49], 2021	−0.69	−1.81	0.44	.23	98.11	−0.06
Zhu et al [45], 2018	−0.42	−1.43	0.60	0.42	98.40	−0.05

a SMD: standardized mean difference.

## Discussion

### Overview

This systematic review and meta-analysis of DMHIs for patients with cancer yielded 3 key findings. First, the included studies demonstrated considerable diversity in intervention design, ranging from mindfulness-based programs to peer support platforms, yet lacked consistency in theoretical foundations and implementation protocols. Second, while meta-analytic results did not show statistically significant overall effects on anxiety or depression, subgroup analyses revealed that intervention characteristics such as duration and delivery format may influence outcomes. Notably, digital interventions demonstrated differential effects based on duration, with sub-1-month interventions showing efficacy for anxiety and 1‐2 months’ interventions proving particularly effective for depression, highlighting the value of duration-specific treatment protocols. Third, the evidence base was limited by methodological heterogeneity influenced by the variety of study characteristics, and 1 study [44], with most trials exhibiting some degree of bias in their design or reporting. These findings highlight both the potential of digital interventions to address mental health needs in cancer care and the necessity for more standardized, high-quality research to establish their clinical effectiveness and optimal implementation strategies.

### Principal Results

Our primary meta-analysis demonstrated no statistically significant effects of digital interventions on depression or anxiety relative to control conditions. This result, along with the high heterogeneity discovered, highlighted the complexity of delivering scalable mental health support in the population of patients with cancer and the challenges inherent in synthesizing heterogeneous intervention approaches, as well as the need for more nuanced implementation strategies rather than universally applicable ones. These findings extend prior research on DMHIs in oncology populations by providing robust evidence that advances earlier systematic reviews [14,17]. While such reviews identified emerging trends in digital mental health applications for patients with cancer, they were unable to substantiate clinical efficacy due to insufficient data. Our results further corroborate the sole meta-analysis available through 2020 [29], which reported only marginal effects, potentially attributable to limited study power at the time.

Our subgroup analyses found important nuances in intervention effectiveness based on duration and delivery format. Specifically, interventions less than 1 month could significantly reduce anxiety, while interventions for 1‐2 months significantly reduce depression symptoms. This finding is consistent with a previous study demonstrating that psychosocial interventions on relaxation, of <6-week intervention duration, <30-minute intervention dose per session, and <4 hours of total time of intervention showed moderate effects, where a short time is necessary for timely and intensive intervention [53]. These results confirm that psychologically grounded interventions, when delivered effectively and promptly, could represent a critical component of holistic cancer treatment [54]. Conversely, the observed timeline for depression interventions aligns with prior findings that app engagement, typically highest at initial download but declining over time, yields minimal effects on depressive symptoms [55]. Therefore, a medium-to-long time interval for depression intervention is recommended, with clinical evidence demonstrating significant improvement in depressed patients following structured, internet-based CBT programs of 6‐8 weeks duration [56-58].

Notably, stakeholder involvement did not significantly influence intervention outcomes. While individual-based approaches demonstrated no statistically significant effect on alleviating depressive symptoms, this aligned with prior evidence showing comparable efficacy between group and individual therapy modalities [59]. These findings may reflect either (1) the inherent complexity of digital therapeutic dynamics, or (2) differential responsiveness of specific symptom domains (eg, negative cognitions vs social withdrawal) to distinct intervention formats. Such nuances may be particularly salient in populations with cancer, where mental health challenges manifest through multifaceted psychopathology [4].

High heterogeneity was observed across studies, reflecting the diverse patient population with varying psychological needs and a broad spectrum of digital interventions. In addition, the sensitivity analysis showed that the substantial heterogeneity observed in our meta-analysis was influenced by a single study [44]. This study implemented a Cyclic Adjustment Training intervention delivered through WeChat with remarkably frequent touchpoints, weekly dynamic cycles involving continuous nurse-patient and patient-peer communication throughout the adjustment and reintrospection stages. Unlike other digital interventions in our review that typically provided static content or periodic reminders, the study’s approach created a highly interactive, real-time support system with immediate professional oversight and peer engagement [44]. This methodological divergence, combined with the study’s focus on newly diagnosed patients preparing for surgery (rather than those in various treatment phases), likely contributed to the observed effect size variations and explains why sensitivity analyses showed substantial changes when this study was excluded. The intensive, professionally mediated nature of this intervention may have achieved therapeutic benefits through mechanisms distinct from those of other DMHIs, highlighting the importance of considering intervention intensity and professional involvement as key moderators in future research. Additionally, due to the self-reported nature of mental health documentation, the findings should be interpreted with caution.

### Comparison With Prior Studies

This meta-analysis significantly advances the previous meta-analysis with data collection before 2020 [29] by addressing its key limitations and providing novel insights. While the earlier review reported only marginal overall effects and emphasized that insufficient data on intervention uptake obscured understanding of effectiveness [29], our analysis incorporates 4 years of additional research (2020‐2024), substantially expanding the evidence base with contemporary studies reflecting recent advances in DMHIs. We also provide a systematic investigation of intervention-specific effects in DMHIs for patients with cancer for future implementation suggestions, a gap not addressed previously.

### Implications

The findings carry important implications for both clinical practice and the design of DMHIs. The evidence of this study suggests mixed overall effects of DMHIs on anxiety and depression, with considerable heterogeneity across studies, even if the most influential one was removed. However, the subgroup analysis, especially after removing the particular study, showed low heterogeneity and a significant reduction in anxiety and depression given different types of intervention durations. This finding aligns with the established psychological theories, which suggest anxiety’s acute nature benefits from prompt symptom management [60], whereas depression’s pervasive cognitive patterns necessitate longer therapeutic engagement for meaningful change [61,62]. If validated in future research, the exploratory subgroup findings would suggest that clinicians should potentially move beyond generic approaches and implement tailored, symptom-specific treatments. Future research should focus on (1) conducting larger, more adequately powered studies with specific intervention content and types to reduce heterogeneity; (2) standardizing outcome measurement tools; and (3) investigating how DMHIs can be effectively integrated into existing cancer care pathways through potential duration-dependent effects. These directions would significantly advance both the precision and effectiveness of DMHIs in oncology care.

### Strengths and Limitations

Our study possesses methodological strengths that bolster the validity of its conclusions. As this study is the most comprehensive meta-analysis focusing specifically on DMHIs for patients with cancer, it addresses a critical gap in the literature by systematically evaluating how intervention duration and stakeholder involvement influence outcomes. Though based on a small sample size, this approach has enabled us to identify subtle but important patterns in treatment efficacy, thereby providing valuable insights to guide the future development of DMHIs for this population.

Despite these strengths, several interconnected limitations must be acknowledged. First, significant heterogeneity was observed across the included studies, which was also mentioned by previous studies in this domain [16,29]. This variability may stem from differences in target populations (cancer types and disease stages), diverse digital interventions (ranging from basic text messaging to interactive multimedia applications), differing therapeutic mechanisms (cognitive-based therapy vs psychoeducation), and notably, 1 study [44] that used a uniquely intensive intervention approach. We use SMD to mitigate the heterogeneity caused by different data collection approaches. Second, the heterogeneity combined with small subgroup sample sizes (2‐6 studies per subgroup) limits the statistical power and reliability of our duration-based and stakeholder involvement analyses. Therefore, the subgroup analysis should also be interpreted with caution. In particular, it would be valuable to further explore anxiety and depression by examining variables such as intervention duration. Since the current number of available studies is limited (n=2‐4), this analysis will need to be revisited in the future. Third, additional limitations include the reliance on non-standardized, self-reported outcome measures, which may introduce bias through recall errors, social desirability, or subjective symptom perceptions [63,64]. Several methodological limitations also warrant consideration. Our restriction to English-language publications may have introduced language bias, potentially excluding relevant studies from non-English speaking populations and limiting generalizability across diverse cultural contexts. The exclusive focus on RCTs, while ensuring methodological rigor, may have excluded valuable insights from quasi-experimental studies or real-world implementation data. Additionally, the rapid pace of technological advancement in digital health creates inherent challenges, as interventions evaluated in older studies may not reflect current technological capabilities or user expectations. The emerging nature of this field means that the evidence base, while growing, remains limited. Many of the interventions evaluated were developed using different theoretical frameworks and technological platforms, contributing to the observed heterogeneity. As DMHIs continue to evolve and mature as therapeutic tools, given the problem of high heterogeneity and one-size-fits-all intervention designs in this realm observed [16,29], a critical need is necessary for incorporating more contemporary RCTs with standardized outcomes to strengthen the evidence base and yield more definitive conclusions. Until such evidence becomes available, the subgroup findings presented here should be considered exploratory and interpreted with appropriate caution.

### Conclusions

While DMHIs overall did not demonstrate significant effects on anxiety or depression with the large study heterogeneity, targeted approaches, particularly short-term interventions for anxiety and depression, showed consistent, promising results across studies. These preliminary findings highlight the importance of tailoring DMHI support to specific patient needs, though they should be interpreted with caution due to the small sample size. As digital interventions continue to evolve in cancer care, greater attention to more methodologically rigorous studies and intervention timing, format, and personalization will be essential to maximize their therapeutic potential.

## Supplementary material

10.2196/64754 Multimedia Appendix 1 PRISMA guideline.

10.2196/64754 Multimedia Appendix 2 Searching strategy summary.

10.2196/64754 Multimedia Appendix 3 Study characteristics summary in the review.

10.2196/64754 Multimedia Appendix 4 Data extraction in meta-analysis.

## Abbreviations

CBT: cognitive behavior therapy
DMHI: digital mental health intervention
PRISMA: Preferred Reporting Items for Systematic Reviews and Meta-Analyses
RCT: Randomized controlled trial
RoB 2: Version 2 of the Cochrane risk-of-bias tool for randomized trials
SF-36: Items Short Form Self Survey
SF12: Short-form 12-item health survey questionnaire
SF36: Short-form 36-item health survey questionnaire
