# Supplementary material for: Digital Interventions and Mental Health Outcomes in Patients With Cancer: Systematic Review and Meta-Analysis
Source: JMIR Cancer. 2025 Aug 14;11:e64754. doi: 10.2196/64754 (PMC12352589; doi:10.2196/64754)
Supplement: Multimedia Appendix 2 [file cancer-v11-e64754-s002.docx]

**Supplement: Search Strategies**

**PubMed:** ("digital intervention"[tiab] OR "mHealth"[tiab] OR "eHealth"[tiab] OR "telehealth"[tiab] OR "web-based"[tiab] OR "smartphone"[tiab] OR "online"[tiab] OR "mobile application"[tiab] OR "digital platform"[tiab] OR "digital therapy"[tiab] OR "internet-based intervention"[tiab] OR "computer-based intervention"[tiab]) AND ("cancer"[tiab] OR "oncology"[tiab] OR "tumor patients"[tiab] OR "neoplasm"[tiab] OR "malignancy"[tiab] OR "cancer survivors"[tiab]) AND ("mental health"[tiab] OR "psychological"[tiab] OR "depression"[tiab] OR "anxiety"[tiab] OR "distress"[tiab] OR "stress"[tiab] OR "emotional well-being"[tiab] OR "quality of life"[tiab] OR "mood"[tiab] OR "psychological functioning"[tiab] OR "resilience"[tiab] OR "self-efficacy"[tiab] OR "coping"[tiab]) AND (English[lang] AND "adult"[MeSH])

**MEDLINE:** ("digital intervention" OR "mHealth" OR "eHealth" OR "telehealth" OR "web-based intervention" OR "smartphone application" OR "online therapy" OR "mobile health" OR "digital mental health").mp. AND ("cancer patients" OR "oncology patients" OR "tumor patients" OR "cancer survivors").mp. AND ("mental health outcomes" OR "psychological outcomes" OR "depression" OR "anxiety" OR "emotional functioning" OR "quality of life" OR "psychological well-being" OR "distress management").mp.

**Web of Science:** TS=("digital intervention" OR "mHealth" OR "eHealth" OR "telehealth" OR "web-based" OR "smartphone" OR "online intervention" OR "digital platform") AND TS=("cancer" OR "oncology" OR "tumor" OR "neoplasm") AND TS=("mental health" OR "psychological" OR "depression" OR "anxiety" OR "quality of life" OR "emotional well-being" OR "psychological functioning" OR "distress" OR "mood disorders")

**Scopus:** TITLE-ABS-KEY("digital intervention" OR "mHealth" OR "eHealth" OR "telehealth" OR "web-based intervention" OR "smartphone application" OR "online therapy" OR "digital mental health intervention") AND TITLE-ABS-KEY("cancer patients" OR "oncology population" OR "tumor patients") AND TITLE-ABS-KEY("mental health outcomes" OR "psychological outcomes" OR "depression" OR "anxiety" OR "quality of life" OR "emotional well-being" OR "psychological distress")

**PsycINFO:** ab("digital intervention" OR "mHealth" OR "eHealth" OR "telehealth" OR "web-based therapy" OR "online intervention" OR "smartphone application" OR "digital mental health" OR "psychological intervention") AND ab("cancer patients" OR "oncology patients") AND ab("mental health" OR "psychological outcomes" OR "depression" OR "anxiety" OR "quality of life" OR "emotional functioning" OR "psychological well-being")

**Global Health:** ("digital intervention" OR "mHealth" OR "eHealth" OR "telehealth" OR "web-based intervention" OR "mobile health" OR "online therapy" OR "digital platform").mp. AND ("cancer" OR "oncology" OR "tumor patients").mp. AND ("mental health" OR "psychological health" OR "depression" OR "anxiety" OR "quality of life" OR "emotional well-being" OR "psychological functioning").mp.

**Embase:** ('digital intervention'/exp OR 'mhealth'/exp OR 'ehealth'/exp OR 'telehealth'/exp OR 'web-based intervention'/exp OR 'smartphone application'/exp OR 'online therapy'/exp OR 'digital mental health intervention'/exp) AND ('cancer patients'/exp OR 'oncology patients'/exp) AND ('mental health outcomes'/exp OR 'depression'/exp OR 'anxiety'/exp OR 'quality of life'/exp OR 'psychological well-being'/exp) AND [english]/lim

**CENTRAL (Cochrane):** #1 "digital intervention" OR "mHealth" OR "eHealth" OR "telehealth" OR "web-based intervention" OR "smartphone application" OR "online therapy" OR "mobile health intervention" OR "digital platform" #2 "cancer patients" OR "oncology patients" OR "tumor patients"
#3 "mental health" OR "psychological outcomes" OR "depression" OR "anxiety" OR "quality of life" OR "emotional functioning" OR "psychological distress" OR "mood" OR "resilience" #4 #1 AND #2 AND #3
